# The functional neuroanatomy of emotion processing in frontotemporal dementias

**DOI:** 10.1093/brain/awz204

**Published:** 2019-07-18

**Authors:** Charles R Marshall, Christopher J D Hardy, Lucy L Russell, Rebecca L Bond, Harri Sivasathiaseelan, Caroline Greaves, Katrina M Moore, Jennifer L Agustus, Janneke E P van Leeuwen, Stephen J Wastling, Jonathan D Rohrer, James M Kilner, Jason D Warren

**Affiliations:** 1 Preventive Neurology Unit, Wolfson Institute of Preventive Medicine, Queen Mary University of London, London, UK; 2 Dementia Research Centre, Department of Neurodegenerative Disease, UCL Institute of Neurology, London, UK; 3 Sobell Department of Motor Neuroscience and Movement Disorders, UCL Institute of Neurology, London, UK; 4 Department of Neuroradiology, National Hospital for Neurology and Neurosurgery, London, UK

**Keywords:** frontotemporal dementia, functional MRI, emotion, autonomic, cardiac

## Abstract

Impaired processing of emotional signals is a core feature of frontotemporal dementia syndromes, but the underlying neural mechanisms have proved challenging to characterize and measure. Progress in this field may depend on detecting functional changes in the working brain, and disentangling components of emotion processing that include sensory decoding, emotion categorization and emotional contagion. We addressed this using functional MRI of naturalistic, dynamic facial emotion processing with concurrent indices of autonomic arousal, in a cohort of patients representing all major frontotemporal dementia syndromes relative to healthy age-matched individuals. Seventeen patients with behavioural variant frontotemporal dementia [four female; mean (standard deviation) age 64.8 (6.8) years], 12 with semantic variant primary progressive aphasia [four female; 66.9 (7.0) years], nine with non-fluent variant primary progressive aphasia [five female; 67.4 (8.1) years] and 22 healthy controls [12 female; 68.6 (6.8) years] passively viewed videos of universal facial expressions during functional MRI acquisition, with simultaneous heart rate and pupillometric recordings; emotion identification accuracy was assessed in a post-scan behavioural task. Relative to healthy controls, patient groups showed significant impairments (analysis of variance models, all *P < *0.05) of facial emotion identification (all syndromes) and cardiac (all syndromes) and pupillary (non-fluent variant only) reactivity. Group-level functional neuroanatomical changes were assessed using statistical parametric mapping, thresholded at *P < *0.05 after correction for multiple comparisons over the whole brain or within pre-specified regions of interest. In response to viewing facial expressions, all participant groups showed comparable activation of primary visual cortex while patient groups showed differential hypo-activation of fusiform and posterior temporo-occipital junctional cortices. Bi-hemispheric, syndrome-specific activations predicting facial emotion identification performance were identified (behavioural variant, anterior insula and caudate; semantic variant, anterior temporal cortex; non-fluent variant, frontal operculum). The semantic and non-fluent variant groups additionally showed complex profiles of central parasympathetic and sympathetic autonomic involvement that overlapped signatures of emotional visual and categorization processing and extended (in the non-fluent group) to brainstem effector pathways. These findings open a window on the functional cerebral mechanisms underpinning complex socio-emotional phenotypes of frontotemporal dementia, with implications for novel physiological biomarker development.

## Teaser

Social and emotional changes are key features of frontotemporal dementia but remain challenging to characterise and measure. Marshall *et al.* report the functional neuroanatomy of aberrant emotion processing across the spectrum of frontotemporal dementia, demonstrating abnormalities at multiple hierarchical levels including sensory processing, emotion categorisation and autonomic reactivity.

## Introduction

Impaired responses to emotional signals are a striking feature of the frontotemporal dementias (FTD) and profoundly disrupt social functioning in these diseases (Rohrer *et al.*, 2012; Hsieh *et al.*, 2013; Warren *et al.*, 2013*a*; Marshall *et al.*, 2018*c*). In the healthy brain, processing of socio-emotional signals such as facial expressions engages four principal, large-scale and hierarchically organized neural networks (Alcalá-López *et al.*, 2017): a ‘visual-sensory’ network of face and biological motion-responsive areas, mediating analysis of stimulus features; a ‘limbic’ network of mesial temporal and ventromedial prefrontal structures, mediating affective valuation of stimuli; an ‘intermediate’ fronto-parietal and cingulo-insular network, integrating salient environmental and bodily states; and a ‘higher associative’ network of temporo-parietal junctional, temporal polar and dorsomedial prefrontal cortices, engaged in interpreting and responding to mental states. Classical models of face processing (Bruce and Young, 1986; Hutchings *et al.*, 2017) map onto these networks, with fractionated systems subserving initial perceptual encoding of faces, subsequent identification of face identity and emotional expression and programming of an appropriate behavioural response. Autonomic reactivity to viewing facial emotions in health engages both visual association areas and the central autonomic control network, including anterior cingulate and insula (Critchley *et al.*, 2005). Targeting of similar brain networks by the proteinopathies of FTD leads, predictably, to diverse socio-emotional symptoms: deficits of face recognition, emotional categorization, motoric and autonomic reactivity and emotional theory of mind have all been demonstrated in FTD and attributed to regional grey matter loss in distributed fronto-temporo-parietal circuitry (Rosen *et al.*, 2002; Kipps and Hodges, 2006; Omar *et al.*, 2011*a*, *b*; Rohrer *et al.*, 2012; Couto *et al.*, 2013; Downey *et al.*, 2013; Oliver *et al.*, 2015; Hazelton *et al.*, 2016; Hutchings *et al.*, 2017; Marshall *et al.*, 2018*a*, *b*). However, the pathophysiological mechanisms that translate neural circuit disintegration to complex socio-emotional phenotypes in these diseases have not been examined directly.

The three major clinico-anatomical syndromes of FTD are each associated with characteristic (though overlapping) behavioural phenotypes and signature atrophy profiles. Considering these profiles in the light of emerging models of the healthy socio-emotional brain (Critchley *et al.*, 2005; Alcalá-López *et al.*, 2017), candidate neural mechanisms of socio-emotional dysfunction in particular FTD syndromes can be proposed. The behavioural variant of FTD (bvFTD) is a heterogeneous syndrome led by changes in social judgment and awareness, with variable profiles of fronto-insular and temporal lobe atrophy (Snowden *et al.*, 2001; Warren *et al.*, 2013*a*). Deficient processing of socio-emotional signals in bvFTD may arise from various levels of the processing hierarchy, encompassing sensory representation, autonomic responses, motor routines, emotional appraisal, and theory of mind (Kipps and Hodges, 2006; Fernandez-Duque *et al.*, 2010; Couto *et al.*, 2013; Baez *et al.*, 2014; Joshi *et al.*, 2014; Cohen *et al.*, 2015; Oliver *et al.*, 2015; Hutchings *et al.*, 2017; Marshall *et al.*, 2018*a*, *b*). Semantic variant primary progressive aphasia (svPPA) is led by multimodal disintegration of semantic knowledge associated with asymmetric, predominantly antero-medial temporal lobe atrophy (Marshall *et al.*, 2018*c*). In svPPA, socio-emotional deficits have been related to erosion of social and emotional concepts and aberrant, overgeneralized or abnormally coupled autonomic and motoric responses to social signals despite retained capacity for emotional reactivity (Fletcher *et al.*, 2015*b*; Marshall *et al.*, 2017, 2018*a*). Non-fluent variant primary progressive aphasia (nfvPPA) is led by breakdown of motor speech output and programming, and is associated with atrophy predominantly affecting left inferior frontal cortex and insula (Marshall *et al.*, 2018*c*). Though typically less prominent than language impairment, socio-emotional deficits are a feature of nfvPPA (Rohrer and Warren, 2010; Hazelton *et al.*, 2016) and may reflect reduced sensorimotor processing of social signals and autonomic arousal (Couto *et al.*, 2013; Cohen *et al.*, 2015; Fletcher *et al.*, 2015*b*; Marshall *et al.*, 2018*a*, *b*).

To establish the pathophysiology of socio-emotional deficits in FTD requires functional neuroanatomical studies that dissect the multiple dimensions of emotion processing. From a clinical perspective, because socio-emotional alterations occur early in the evolution of FTD, improved understanding of the brain dysfunction that underpins these alterations could potentially drive development of new diagnostic and therapeutic biomarkers for disease detection and tracking, prior to the onset of irrecoverable brain damage. Two previous functional MRI studies of facial emotion processing in bvFTD have revealed reduced activity in face-responsive visual cortices, proposed to reflect disrupted top-down influences (Virani *et al.*, 2013; De Winter *et al.*, 2016). However, patterns of neural network dysfunction responsible for socio-emotional symptoms across the FTD spectrum have not yet been elucidated. Moreover, despite mounting evidence that autonomic regulation plays a key role in emotional reactivity in health and in FTD (Marshall *et al.*, 2017, 2018*a*, *b*), the functional neuroanatomy of altered autonomic responses to affective stimuli has not been characterized in these diseases. Relatedly, capturing the pathophysiology of facial emotion processing in FTD is likely to require dynamic stimuli that more closely simulate the naturalistic socio-emotional signals of daily life, rather than conventional static images.

Here we addressed these issues using functional MRI of dynamic facial expressions with simultaneous recording of autonomic (cardiac and pupillary) responses in a cohort of patients representing all major FTD syndromes, and healthy age-matched individuals. Facial expressions were referenced to a comparably complex dynamic, affectively neutral visual baseline and to a simple fixation condition, allowing us to dissect visual sensory from emotion decoding responses. We used a passive viewing functional MRI design with no in-scanner output task to avoid potentially confounding task difficulty or performance monitoring effects; post-scanner behavioural data were collected to assess the accuracy of participants’ emotion identification. Based on available data in the healthy brain and in FTD (Virani *et al.*, 2013; De Winter *et al.*, 2016; Alcalá-López *et al.*, 2017; Hutchings *et al.*, 2017), we hypothesized that visual processing of dynamic facial expressions would be associated with activation of face and biological motion-responsive cortices and that FTD syndromes would be associated with attenuated activation of cortical mechanisms encoding emotions despite normal early visual processing. We further hypothesized that all FTD syndromes would be associated with impaired emotion identification but that syndromes would be differentiated based on their relative involvement of emotion evaluation and categorization mechanisms. Finally, we hypothesized that syndromic profiles of altered cardiac and pupillary reactivity would predict differential engagement of visual association and central autonomic control and effector pathways (Critchley *et al.*, 2005; Marshall *et al.*, 2018*a*, *b*).

## Materials and methods

### Participants

Sixty participants were included, comprising 38 patients fulfilling consensus criteria for a syndrome of FTD (Gorno-Tempini *et al.*, 2011; Rascovsky *et al.*, 2011) (17 with bvFTD, 12 with svPPA, nine with nfvPPA) of mild to moderate severity and 22 healthy older individuals with no history of neurological or psychiatric illness. No participant had a significant burden of cerebrovascular disease or visual loss. In all patients the syndromic diagnosis was endorsed by clinical and neuropsychological assessment and volumetric T_1_ brain MRI (see Supplementary Fig. 1 for patient group atrophy profiles). Five bvFTD patients had an identified disease-causing mutation (three *MAPT*, one *C9orf72*, one *GRN*). Demographic, clinical and neuropsychological characteristics of the participant groups are summarized in Table 1.

**Table 1:** Clinical and neuropsychological characteristics of participant groups

**Characteristic**	**Controls**	**bvFTD**	**svPPA**	**nfvPPA**
**Demographic and clinical**				
*n*, male:female	10:12	13:4	8:4	4:5
Age, years	68.6 (6.8)	64.8 (6.8)	66.9 (7.0)	67.4 (8.1)
Handedness, right:left:ambidextrous	22:0	15:1	12:0	8:0
Years of education	16.1 (2.5)	13.9 (5.0)	15.6 (2.7)	13.0 (3.4)
MMSE (/30)	29.8 (0.4)	23.7 (4.8)^a^	23.8 (7.4)^a^	16.9 (10.9)^a^^,b,c^
Duration, years	N/A	7.2 (6.3)	6.0 (2.6)	3.8 (1.7)
**General neuropsychological**				
General intellect				
WASI verbal IQ	122 (8.6)	92 (31.5)^a^	74 (20.1)^a^	69 (17.7)^a^
WASI performance IQ	124 (12.9)	96 (18.3)^a^^,c^	119 (15.4)	94 (20.8)^a^^,c^
Episodic memory				
RMT words (/50)	48.9 (1.4)	37.6 (10.2)^a^	33.8 (7.3)^a^	39.2 (10.8)^a^
RMT faces (/50)	44.8 (4.7)	37.3 (7.0)^a^	32.1 (5.0)^a^	39.0 (7.9)
Camden PAL (/24)	20.6 (2.8)	13.7 (6.1)^a^	6.5 (8.0)^a^^,b,d^	16.5 (2.1)
Executive skills				
WASI Block Design (/71)	46.8 (11.0)	26.9 (15.1)^a^	38.5 (15.6)	20.5 (20.5)^a^
WASI Matrices (/32)	25.5 (4.4)	16.7 (8.7)^a^^,c^	26.6 (3.5)	15.4 (10.2)^a^^,c^
WMS-R digit span forward (max)	7.1 (1.1)	5.7 (1.1)^a^	6.6 (0.9)	4.3 (1.4)^a^^,c^
WMS-R digit span reverse (max)	5.4 (1.3)	4.6 (1.4)	5.3 (1.3)	3.2 (0.8)^a^^,c^
D-KEFS Stroop colour naming (s)	29.6 (4.8)	45.3 (19.5)^a^	37.8 (8.9)	70.0 (18.7)^a^^,b,c^
D-KEFS Stroop word reading (s)	22.3 (3.4)	28.2 (7.5)	25.6 (10.7)	61.4 (16.2)^a^^,b,c^
D-KEFS Stroop interference (s)	55.9 (16.7)	101.1 (52.6)^a^	67.3 (19.0)	123.3 (44.3)^a^^,c^
Letter fluency (F: total)	17.4 (5.0)	9.0 (5.6)^a^	9.6 (3.8)^a^	5.8 (3.3)^a^
Category fluency (animals: total)	23.7 (4.2)	13.0 (8.0)^a^	6.5 (4.5)^a^^,b^	12.6 (4.7)^a^
Trails A (s)	31.9 (9.3)	58.1 (36.3)^a^	46.7 (16.1)	65.3 (45.4)^a^
Trails B (s)	66.3 (28.6)	143.7 (81.6)^a^	130.5 (18.8)^a^	160.1 (89.7)^a^
Language skills				
WASI vocabulary	70.3 (3.4)	40.9 (24.8)^a^	30.6 (18.9)^a^	21.8 (21.3)^a^
BPVS	148.0 (1.4)	126.2 (30.6)^a^	74.8 (37.1)^a^^,b^	106.4 (52.8)^a^
GNT	26.9 (2.3)	16.7 (10.2)^a^	2.0 (5.6)^a^^,b^	9.0 (7.3)^a^
Other skills				
GDA (/24)	14.1 (5.4)	9.3 (6.1)	12.8 (5.0)	4.8 (5.1)^a^
VOSP Object Decision (/20)	18.9 (1.1)	15.7 (3.4)^a^	15.9 (2.0)^a^	15.5 (3.9)^a^
**Emotion identification^e^**				
Anger	4.43 (1.9)	3.31 (2.0)	2.50 (2.0)	3.88 (1.5)
Disgust	8.81 (1.1)	6.13 (2.9)^a^	5.33 (2.0)^a^	5.00 (3.9)^a^
Fear	5.48 (2.4)	3.69 (2.9)	3.42 (2.2)	4.88 (2.9)
Happiness	9.43 (0.8)	8.44 (2.3)	9.08 (0.9)	7.13 (3.1)
Surprise	7.76 (1.4)	5.00 (3.2)^a^	3.75 (2.5)^a^	4.00 (3.3)^a^
Total (/50)	35.9 (4.1)	26.6 (9.5)^a^	24.1 (5.7)^a^	24.9 (12.6)^a^

Mean (standard deviation) scores are shown unless otherwise indicated; maximum scores are shown after tests (in parentheses).

^a^Significantly less than controls; ^b^significantly less than bvFTD; ^c^significantly less than svPPA; ^d^significantly less than nfvPPA, (all *P* < 0.05).

^e^Post-scanner experimental test of facial expression identification (see main text and Fig. 3).

BPVS = British Picture Vocabulary Scale (Dunn and Whetton, 1982); Category fluency totals for animal category and letter fluency for the letter F in 1 min (Gladsjo *et al.*, 1999); D-KEFS = Delis Kaplan Executive System (Delis *et al.*, 2001); GDA = Graded Difficulty Arithmetic (Jackson and Warrington, 1986); GNT = Graded Naming Test (McKenna and Warrington, 1980); MMSE = Mini-Mental State Examination score (Folstein *et al.*, 1975); N/A = not assessed; PAL = Paired Associate Learning test (Warrington, 1996); RMT = Recognition Memory Test (Warrington, 1984); Trails-making task based on maximum time achievable 2.5 min on task A, 5 min on task B (Lezak, 2004); VOSP = Visual Object and Spatial Perception Battery – Object Decision test (Warrington and James, 1991); WASI = Wechsler Abbreviated Scale of Intelligence (Wechsler, 1997); WMS = Wechsler Memory Scale (Wechsler, 1987).

This study was approved by the University College London institutional ethics committee and all participants gave informed consent in accordance with the Declaration of Helsinki.

### Experimental stimuli

Videos of dynamic emotional facial expressions were obtained from the Face and Gesture Recognition Research Network (FG-NET) database (Wallhoff, 2006–2015). This database comprises silent recordings of young adults viewing emotional scenarios, designed to elicit spontaneous, naturalistic facial expressions but presented without any instruction to pose particular expressions. For each of the canonical emotions of anger, disgust, fear, happiness and surprise (Ekman *et al.*, 1969) we selected 10 videos (50 stimuli in total) that clearly conveyed the relevant expression (sadness was omitted because its more diffuse time course sets it apart from other emotional expressions). Each video stimulus lasted between 4 and 8 s (mean 4.9 s), commencing as a neutral facial expression and evolving into an emotional expression. We did not include a neutral face condition because so-called ‘neutral’ faces are often interpreted as displaying negative affect (Rich *et al.*, 2006; Suess *et al.*, 2014). Using dynamic stimuli would tend to exaggerate this effect: in that context, an immobile face would appear hostile, while facial muscle movements not included in canonical emotional expressions nevertheless frequently transmit emotional content (Wallbott and Ricci-Bitti, 1993). For this reason, other studies of dynamic facial emotions have often used an abstract visual baseline (Grosbras and Paus, 2005; Sato *et al.*, 2015). To provide a complex visual baseline without facial emotion features, we created 20 dynamic mosaics from the videos by dividing each video frame into 400 equal rectangles (20 × 20), and then randomizing the position of the rectangles within each video (the positions then remained consistent across all frames for a given stimulus). These dynamic mosaics were thus matched with the original videos for luminance, colour, contrast, motion, and duration, but without discernible face or emotional content, i.e. the same physical information was present, but the global configuration was radically altered.

### Stimulus presentation

During functional MRI scanning, stimuli were presented in a pseudorandomized block design (five stimuli per block) via a notebook computer using Eyelink Experiment Builder software (SR-Research, Ottawa, Canada). Each stimulus trial was triggered by the magnetic resonance scanner at the onset of a gradient echo-echo planar imaging (GE-EPI) volume acquisition. Visual stimuli were presented on a screen placed outside the bore of the MRI scanner, visible to participants in a periscopic mirror affixed to the radiofrequency (RF) head coil. A total of 90 trials were delivered, comprising 50 dynamic facial stimuli, 20 dynamic scrambled visual mosaics and 20 fixation cross trials (to allow estimation of primary visual sensory processing). Interstimulus interval was 11.72 s for video trials and 8.79 s for fixation cross trials. Following the end of each stimulus, a grey screen was presented until the onset of the next trial. To avoid potentially confounding effects from task preparation, difficulty and performance monitoring, participants were simply instructed to lie still and concentrate on the stimuli with their eyes open; no responses from the participants were obtained during scanning. All participants were remotely monitored via an MRI-compatible Eyelink 1000Plus eyetracker (SR-Research) to ensure they had their eyes open and were fixating on the stimuli.

### Brain MRI acquisition

Functional MRI data were acquired using a 3 T Siemens Prisma scanner with a 12-channel RF head coil. A continuous acquisition GE-EPI sequence was used comprising 48 oblique axial slices covering the whole brain. The angle of acquisition was set at −30° from the intercomissural plane to minimize susceptibility-induced signal dropout in orbitofrontal cortex and anterior temporal lobes because of the proximity of these regions to the skull base. Interleaved slices of 2-mm thickness were obtained in descending order with voxel size 2 × 2 × 2 mm, field of view 192 mm, repetition time 2930 ms and echo time 30 ms. For each participant, 340 EPI volumes covering all 90 stimulus presentation trials were obtained for analysis (four volumes for each video trial, and three for each fixation cross trial), with a total scanning time of 16 min 40 s. Following acquisition of the functional MRI scan, a B0 field map was acquired to allow geometric correction of EPI data for field inhomogeneity distortions (field of view 192 mm, slice thickness 3 mm interleaved, voxel size 2.4 × 2.4 × 3 mm, repetition time 688 ms, echo time 1 4.92 ms, echo time 2 7.38 ms).

To enable structural co-registration of functional MRI data, volumetric brain MRIs were acquired for all patients in the same 3 T Siemens Prisma MRI scanner, using a 64-channel head-and-neck RF coil with a T_1_-weighted sagittal 3D magnetization prepared rapid gradient echo (MPRAGE) sequence (echo time = 2.93 ms, inversion time = 850 ms, repetition time = 2000 ms), with matrix size 256 × 256 × 208 and voxel dimensions 1.1 × 1.1 × 1.1 mm. Parallel imaging (GRAPPA) was used with acceleration factor 2, resulting in an overall scan time of 5 min 6 s.

### Autonomic recordings

Simultaneously with functional MRI data acquisition, heart rate was recorded continuously from the left index finger during scanning using an MRI-compatible pulse oximeter (Siemens, Erlangen, Germany). Pulse oximetry is typically the modality of choice for in-scanner heart rate measurement due to problems with scanner artefact in ECG recordings (Critchley *et al.*, 2005; Guo *et al.*, 2016). In addition, a long-range mount positioned within the bore of the scanner captured the participant’s right eye in the periscopic mirror; pupil size was recorded throughout scanning using the eyetracker.

### Post-scan behavioural testing

Following the scanning session, each participant was shown the 50 facial emotion stimuli presented during scanning, using the Eyelink Experiment Builder software package on a notebook computer. After each video, they were asked to identify the emotion from a list of the five emotions used in the experiment. Responses were recorded for offline analysis. No time limits were imposed on responses, and no feedback was given during the task.

### Analysis of autonomic and behavioural data

Raw heart rate data were analysed offline in MATLAB using a custom script to identify local maxima corresponding to pulse peaks in the waveform. All data were manually inspected to ensure consistency and accuracy of pulse detection. Data from participants with cardiac arrhythmias (e.g. atrial fibrillation) or of insufficient quality were excluded from subsequent heart rate analyses (three healthy controls, four patients with bvFTD, two with svPPA and one with nfvPPA). For each participant, a continuous smoothed heart rate trace was generated by converting each data point to the heart rate corresponding to the inter-beat interval in which it occurred, and then smoothing with a 1-s sliding filter. A heart rate reactivity trace was then generated for each trial by normalizing to the baseline heart rate for that trial, so that all values represented percentage heart rate change from trial baseline. Heart rate change was analysed across eight time-bins at 500-ms intervals from 0.5 s to 4 s from stimulus onset. This heart rate reactivity measure was analysed as the dependent variable in an ANOVA model, incorporating stimulus type and diagnostic group as fixed factors. *Post hoc* tests with Bonferroni correction were performed when main effects were found. Visualization of the mean trial heart rate trace for healthy controls showed that there was a consistent cardiac deceleration, with a nadir between 3 and 4 s from stimulus onset (Supplementary Fig. 2). A mean heart rate reactivity measure for each participant was therefore defined as the mean change in heart rate from baseline at 3 s from stimulus onset, and this value was entered into the second-level functional MRI analysis to establish the neural basis for between-participant variance in heart rate reactivity.

Pupillometry data were analysed offline using the SR Research Data Viewer software. Pupil reactivity was calculated for each trial as follows:

100 × max pupil size during 5 s post stimulus onset / mean pupil size during 1 s prior to stimulus onset (1)

Trials with pupil reactivity values over two standard deviations above the experimental mean (and therefore potentially contaminated by large artefacts, e.g. blinks) and trials with insufficient pupil capture were removed; overall, 17% of trials were excluded from subsequent analysis. Pupil reactivity was analysed for facial emotion and scrambled videos, but not for fixation cross trials, as the large difference in luminance between the video conditions and fixation cross conditions made them unsuitable for direct comparison. An ANOVA model was used to assess main effects on pupil size change of participant group, stimulus condition type and the interaction between the two. *Post hoc* tests with Bonferroni correction were performed when significant main effects were found. Mean pupil reactivity for each participant was entered into the second-level functional MRI analysis to establish the neural basis for between-participant variance in pupil reactivity.

Emotion identification scores were compared among groups using an ANOVA model, with Bonferroni-corrected *post hoc t*-tests when main effects were found. To explore the effect of deficits in other cognitive domains on emotion identification ability, cardiac reactivity and pupil reactivity, correlations were tested between these parameters and performance on tests of working memory (forward digit span), non-verbal intelligence (WASI Matrices), general executive function (Trail-making B test) and semantic knowledge (British Picture Vocabulary Scale).

A statistical threshold *P < *0.05 (Bonferroni-corrected where appropriate for *post hoc* multiple comparisons) was accepted for all tests.

### Preprocessing and analysis of functional MRI data

Functional MRI data were processed using SPM12 software (www.fil.ion.ucl.ac.uk/spm) in MATLAB R2014b. The EPI series for each participant was realigned to the first image and unwarped with incorporation of B0 distortion information to correct for field inhomogeneities. The T_1_ volumetric image for each participant was registered to their EPI images before segmentation into grey matter, white matter and CSF using the New Segment toolbox of SPM. Forward deformations from the segmentation step were then used to normalize the EPI images into MNI space before smoothing the normalized unwarped EPI images with a 6 mm full-width at half-maximum Gaussian kernel. Each registration and normalization step was visually checked for quality control; in five participants, preprocessing was repeated with an additional skull-stripping step prior to registration.

Preprocessed GE-EPI images were entered into a first-level analysis for each participant incorporating the experimental conditions as separate regressors, modelled as a boxcar across the duration of each individual trial, and convolved with the canonical haemodynamic response function. Six head motion parameters were included as covariates of no interest. A liberal masking threshold of 0.1 was used at first level, to ensure that regions showing atrophy in some participants were not entirely excluded from the second-level analysis, where a majority threshold mask was applied (see Supplementary material for more detail on preprocessing performance in the presence of atrophy). T contrasts between conditions were generated from the first-level analysis: the contrast of facial emotion > fixation cross conditions was used to assess sensory processing of dynamic facial expressions, and the contrast of facial emotion > scrambled video conditions was used to assess decoding of facial emotions. The contrasts of positive facial emotion > negative facial emotion and negative facial emotion > positive facial emotion were used to assess valence-specific activation patterns (happiness and surprise were defined as positive emotions, anger, disgust and fear were defined as negative emotions.

In the second-level analysis, T contrasts from the first-level analysis were entered into a full factorial model incorporating all participants, with diagnostic group as a level variable. Masking was performed with a study-specific majority threshold mask (Ridgway *et al.*, 2009). The effects of experimental conditions were modelled by assessing T contrasts for effect of condition across all participants, and F contrasts to detect group differences. Where main effects of participant group were found in the F contrast, group differences were assessed by generating beta plots incorporating all voxels in the relevant cluster. Beta plots for primary visual cortex were also generated to examine whether there were any between-group differences in primary afferent processing.

To establish the neural basis for between-participant differences in emotion identification ability and autonomic responses, total emotion identification score or mean physiological response parameter for each participant was incorporated as a second-level covariate, assessing T contrasts within each participant group separately to establish haemodynamic responses that explained variance in these parameters within each disease group (i.e. syndromic-specific predictors of response rather than activation differences between groups). For emotion identification ability, British Picture Vocabulary Scale scores for each participant were included as a covariate to remove variance attributable to semantic deficits. For cardiac responses, both negative (parasympathetic) and positive (sympathetic) correlations with heart rate change were assessed (Beissner *et al.*, 2013). Although the precise neural inputs responsible for heart rate changes could not be measured (e.g. cardiac acceleration could be due to increased sympathetic input or decreased parasympathetic input), we used cardiac acceleration as a proxy for an overall shift in favour of sympathetic tone and vice versa (Paulus *et al.*, 2016).

For all functional MRI analyses, we applied a cluster-defining uncorrected significance threshold *P < *0.005; this cluster-defining threshold was selected according to evidence that it provides the optimal balance between the risks of type I and type II errors (Lieberman and Cunningham, 2009). The significance of blood oxygen level-dependent (BOLD) changes was assessed at two thresholds: at cluster level *P < *0.05, after family-wise error (FWE) correction for multiple comparisons over the whole brain; and at peak voxel level *P*_FWE_ < 0.05 within pre-specified anatomical regions of interest. These thresholds are complementary, allowing detection of robust, potentially novel associations (over the whole brain) while also incorporating prior hypotheses about likely regional associations, informed by previous work. Anatomical regions of interest were defined separately for each analysis based on previous evidence in the healthy brain and in FTD cohorts: for sensory processing of dynamic facial expressions, this region comprised fusiform face area, MT/V5, posterior superior temporal sulcus and middle temporal gyrus (Haxby and Gobbini, 2011; Alcalá-López *et al.*, 2017); for identification of facial emotions, fusiform gyrus, anterior cingulate, insula, frontal operculum and anteromedial temporal lobe (Zahn *et al.*, 2007; Jabbi and Keysers, 2008; Alcalá-López *et al.*, 2017); and for autonomic reactivity, fusiform gyrus, anteromedial temporal lobe, anterior cingulate and insula (Critchley *et al.*, 2005; Beissner *et al.*, 2013; Cersosimo and Benarroch, 2013; Fletcher *et al.*, 2016).

A study-specific mean brain image generated from all participants’ normalized T_1_ MRIs was used to display SPM results thresholded at uncorrected threshold *P < *0.005 for display purposes.

### Data availability

The data that support the findings of this study are available on request from the corresponding author. The data are not publicly available as they include information that could compromise the privacy of the research participants.

## Results

### General characteristics of participant groups

Participant groups did not differ significantly in age, gender or years of education (suggesting they were likely to be well matched for premorbid IQ), and patient groups had similar symptom durations.

### Identification of facial emotions

Performance data for the post-scan emotion identification task are presented in Table 1 and Fig. 3. There were main effects of participant group [*F*(3) = 49.9, *P < *0.001] and emotion type [*F*(4) = 26.0, *P < *0.001], but no significant interaction between group and emotion [*F*(12) = 1.55, *P* = 0.10]. *Post hoc* tests demonstrated impaired emotion identification in all disease groups relative to healthy controls (all *P*_Bonf_ < 0.001) and in the svPPA group relative to the bvFTD group (*P*_Bonf_ = 0.038). Across the combined participant cohort, identification scores were higher for disgust and happiness than for other emotions (all pairwise comparisons *P*_Bonf_ < 0.001); while scores for anger identification were lower than those for fear (*P*_Bonf_ = 0.046) and surprise (*P*_Bonf_ = 0.012). Overall emotion identification ability correlated significantly with working memory (forward digit span; *P = *0.002), general executive function (Trail-making B score; *P < *0.001), non-verbal intelligence (WASI Matrices score; *P = *0.001) and semantic competence (British Picture Vocabulary Scale score; *P < *0.001).

### Cardiac reactivity

Participant groups did not differ in mean heart rate during the period of recording [*F*(3) = 1.23, *P = *0.32], nor in overall heart rate variability [indexed as the variance of interbeat intervals; *F*(3) = 0.756, *P = *0.525].

In the healthy control group, a consistent cardiac deceleration was shown for all stimulus conditions (one-sample *t*-test, *P < *0.001). There was a main effect of stimulus condition on cardiac reactivity [*F*(2) = 6.3, *P = *0.002], *post hoc* tests showing that greater cardiac deceleration occurred for emotional facial expressions than scrambled videos (*P*_Bonf_ = 0.033) and fixation crosses (*P*_Bonf_ = 0.009), with no significant difference between scrambled video and fixation cross conditions (*P*_Bonf_ = 1). Considering facial emotions separately, the healthy control group showed a main effect of emotion type on cardiac reactivity [*F*(6) = 11.35, *P < *0.001]. *Post hoc* tests revealed that cardiac deceleration was greater for happiness than other emotions (all individual pairwise comparisons *P*_Bonf_ < 0.001). No other emotion-specific differences were identified in the healthy control group.

Across all participants, cardiac reactivity showed main effects of participant group [*F*(3) = 10.12 *P < *0.001], stimulus type [*F*(6) = 12.89, *P < *0.001] and a significant interaction of group and stimulus type [*F*(18) = 3.21, *P < *0.001]. Relative to healthy controls, cardiac deceleration to visual stimuli was significantly attenuated in each patient group (all *post hoc* pairwise comparisons *P*_Bonf_ < 0.007). There were no significant differences between patient groups (all *P*_Bonf_ > 0.4). Mean cardiac responses to visual stimuli in each participant group are presented in Fig. 4; data for each stimulus type and participant group separately are presented in Supplementary Fig. 3.

There were no significant correlations between cardiac reactivity and neuropsychological measures of working memory, general executive function, non-verbal intelligence or semantic competence (all *P* > 0.3).

### Pupil reactivity

There were main effects on pupil responses to video stimuli from both participant group [*F*(3) = 8.714, *P < *0.001] and stimulus condition [*F*(5) = 3.149, *P = *0.008], but no significant interaction between group and condition [*F*(15) = 0.91, *P = *0.55]. *Post hoc* tests revealed that pupil reactivity was significantly less for scrambled videos than for facial emotions (*P < *0.001), but did not differ between facial emotions (all *P* > 0.08). Relative to healthy controls, pupil responses to visual stimuli were significantly reduced in the nfvPPA group (*P*_Bonf_ < 0.001) but not the svPPA group (*P*_Bonf_ = 0.078) or bvFTD group (*P*_Bonf_ = 1). Mean pupil responses to visual stimuli in each participant group are displayed in Fig. 5; pupillary responses in each stimulus condition are presented in Supplementary Fig. 4.

There were no significant correlations between pupil reactivity and neuropsychological measures of working memory, general executive function, non-verbal intelligence or semantic competence (all *P* > 0.12).

### Functional neuroanatomy

**Figure 1:**
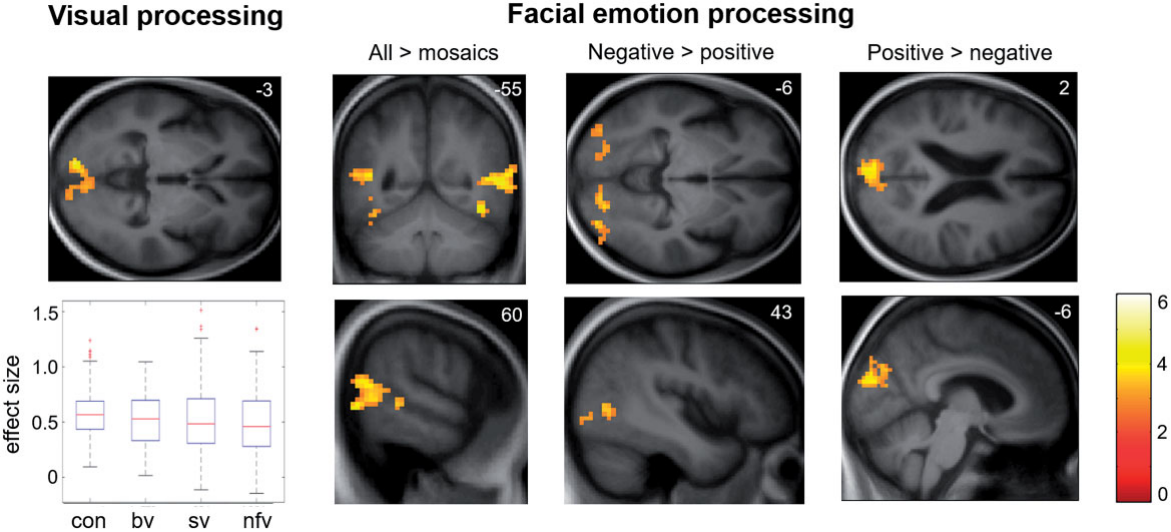
**Functional neuroanatomy of facial emotion viewing: effect of condition.** Statistical parametric maps (SPMs) of T contrasts for effect of condition across all participants for early visual processing (visual stimulus > fixation cross contrast; *left*) and facial emotion processing (contrasts for all facial expressions > dynamic mosaic baseline, positive facial expressions > negative expressions, negative facial expressions > positive expressions) together with a plot (*bottom left*) of effect sizes (beta-values) demonstrating consistent activation of bilateral primary visual cortex across participant groups (box and whisker plots display median, interquartile range, minimum and maximum values, with outliers appearing as red crosses). SPMs are thresholded at the cluster-defining threshold of *P < *0.005 uncorrected and displayed on sections of the structural group mean T_1_-weighted template brain image. The plane of each section (in mm in MNI space) is shown in the *top right* of each image; axial sections show the left hemisphere on the *top* and the coronal section shows the left hemisphere on the *left.* The colour bar codes T-values (the same range applies to SPMs in other figures, unless otherwise indicated). bv = patient group with bvFTD; con = healthy control group; nfv = patient group with nfvPPA; sv = patient group with svPPA.

**Figure 2:**
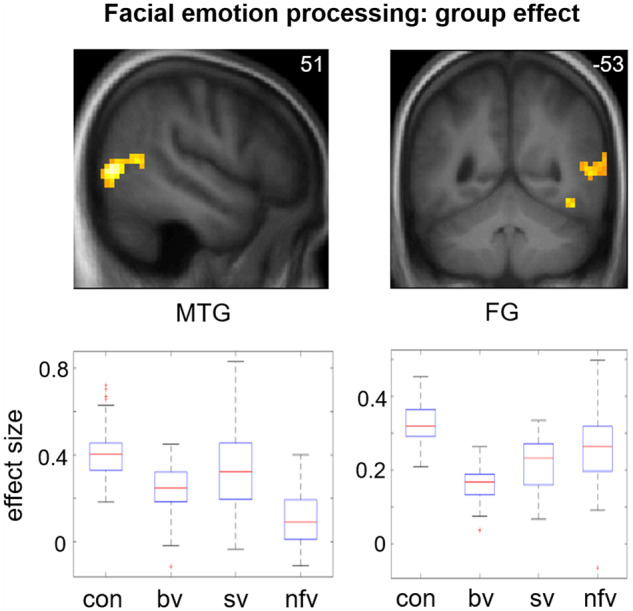
**Functional neuroanatomy of facial emotion viewing: effect of participant group.** Statistical parametric maps (SPMs) for the F-contrast (main effect of participant group; facial emotion > dynamic mosaic contrast; *top row*), together with plots of effect sizes (beta-values) demonstrating differential patterns of attenuated BOLD response across groups in the two significant clusters (*bottom row*; box and whisker plots display median, interquartile range, minimum and maximum values, with outliers appearing as red crosses). SPMs are thresholded at the cluster-defining threshold of *P < *0.005 and displayed on sections of the structural group mean T_1_-weighted template brain image. The plane of each section (in mm in MNI space) is shown in the *top right* of each image; the coronal section shows the right hemisphere on the *right*. bv = patient group with bvFTD; con = healthy control group; FG = fusiform gyrus; MTG = middle temporal gyrus; nfv = patient group with nfvPPA; sv = patient group with svPPA.

**Figure 3:**
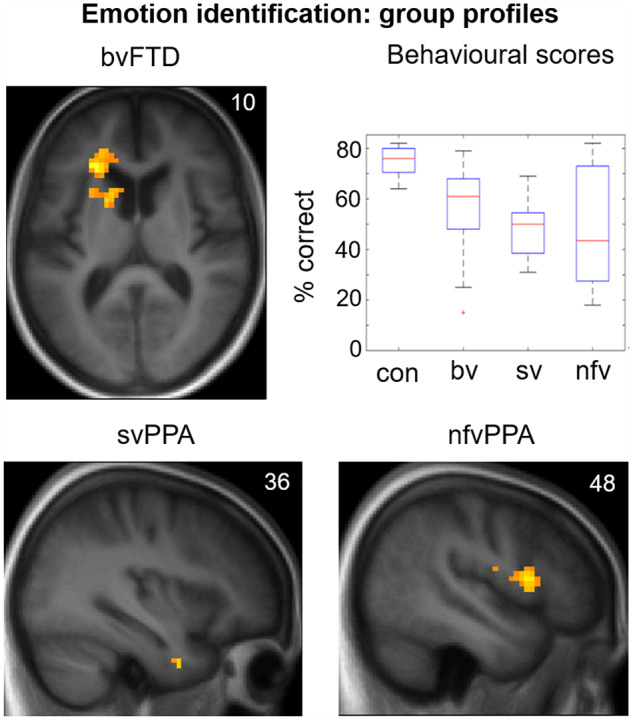
**Emotion identification: behavioural results and functional neuroanatomy.** The figure displays statistical parametric maps (SPMs) for the T-contrast (facial emotion > dynamic mosaic) in each patient group, with score on the post-scanner emotion identification task as predictor variable in order to show the key determinants of identification ability separately within each group (*top left*, *bottom*), together with a plot showing performance (per cent correct) on the emotion identification task by participant group (*top right*; box and whisker plots display median, interquartile range, minimum and maximum values, with outliers appearing as red crosses). SPMs are thresholded at the cluster-defining threshold of *P < *0.005 uncorrected (all loci displayed on the sections shown were significant at *P*_FWE_ < 0.05 at whole brain or in pre-specified regions of interest (see Table 3 for details) and displayed on sections of the structural group mean T_1_-weighted template brain image. The plane of each section (in mm in MNI space) is shown in the *top right* of each image; the axial section shows the left hemisphere on the *left*. bv = patient group with bvFTD; con = healthy control group; nfv = patient group with nfvPPA; sv = patient group with svPPA.

**Figure 4:**
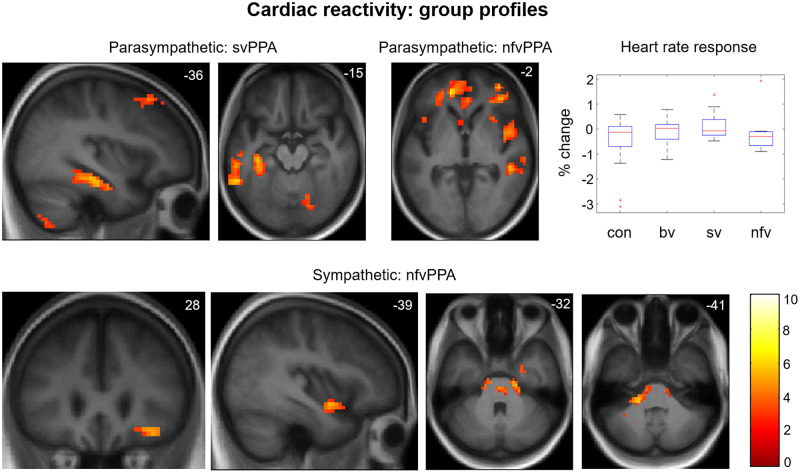
**Cardiac reactivity: heart rate modulation and functional neuroanatomy.** Statistical parametric maps (SPMs) for the T-contrast (facial emotion > dynamic mosaic) in the svPPA and nfvPPA patient groups, with cardiac reactivity as predictor variable. Associations are shown separately for negative correlation with cardiac reactivity (i.e. BOLD signal predicting parasympathetic cardiac deceleration; *top row*) and positive correlation with cardiac reactivity (i.e. BOLD signal predicting sympathetic cardiac acceleration; *bottom row*). The plot (*top right*) shows mean cardiac reactivity (per cent change in heart rate from baseline) to facial expression stimuli by participant group (box and whisker plots display median, interquartile range, minimum and maximum values, with outliers appearing as red crosses). SPMs are thresholded at the cluster-defining threshold of *P < *0.005 uncorrected and displayed on sections of the structural group mean T_1_-weighted template brain image. The plane of each section (in mm in MNI space) is shown in the *top right* of each image; axial sections show the right hemisphere on the *right.* The colour bar codes T-values. bv = patient group with bvFTD; con = healthy control group; nfv = patient group with nfvPPA; sv = patient group with svPPA.

**Figure 5:**
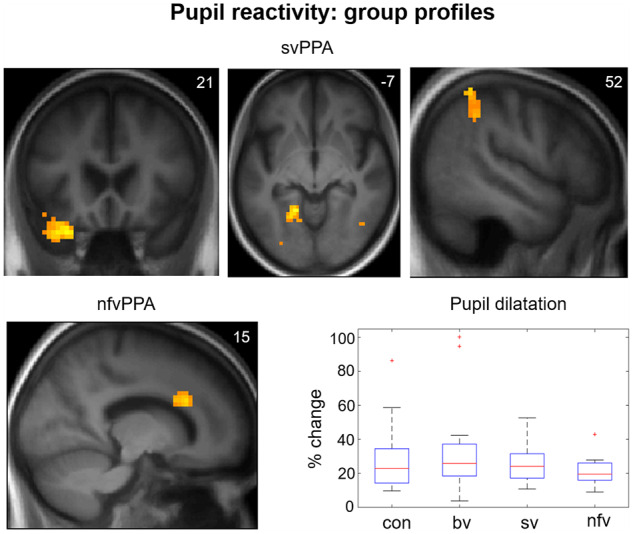
**Pupil reactivity: pupil size change and functional neuroanatomy.** The figure displays statistical parametric maps (SPMs) for the T-contrast (facial emotion > dynamic mosaic) in the svPPA and nfvPPA patient groups, with pupil reactivity as predictor variable. The plot (*bottom right*) shows mean pupil reactivity (per cent increase in pupil size from baseline) to facial expression stimuli by participant group (box and whisker plots display median, interquartile range, minimum and maximum values, with outliers appearing as red crosses). SPMs are thresholded at the cluster-defining threshold of *P < *0.005 uncorrected and displayed on sections of the structural group mean T_1_-weighted template brain image. The plane of each section (in mm in MNI space) is shown in the *top right* of each image; axial and coronal sections show the left hemisphere on the *left*. bv = patient group with bvFTD; con = healthy control group; nfv = patient group with nfvPPA; sv = patient group with svPPA.

Functional neuroanatomical correlates of viewing and identifying facial emotions are shown in Table 2 and Figs 1–3 and correlates of autonomic reactivity are shown in Table 3 and Figs 4 and 5.

**Table 2:** Functional neuroanatomical associations of viewing dynamic facial emotions

**Group**	**Region**	**Side**	**Cluster (voxels)**	**Peak (mm)**			***P*_FWE_**
				***x***	***y***	***z***	
**Early visual processing: effect of condition^a^**							
All	Primary visual cortex	Right	279	15	−94	14	<0.001
		Left	–	−12	91	2	–
**Facial emotion processing: effect of condition^b^**							
All	Area MT/V5	Right	345	51	−70	2	<0.001
	Superior temporal sulcus / middle temporal gyrus	Right	–	57	−34	2	–
	Angular gyrus	Right	–	63	−58	14	–
	Fusiform gyrus	Right	71	42	−46	−16	0.001*
		Left	62	−42	−52	−19	0.021*
	Area MT/V5	Left	87	45	−58	11	0.010*
**Facial emotion processing: positive > negative emotions^c^**							
All	Cuneus	Left	254	−3	−88	23	0.001
	Cuneus	Right	–	6	−82	38	–
**Facial emotion processing: negative > positive emotions^d^**							
All	Inferior occipital gyrus	Right	157	24	−88	2	0.019
	Lingual gyrus	Right	–	15	−91	−4	–
	Fusiform gyrus	Right	–	21	−82	−7	–
	Area MT/V5	Right	25	48	−64	2	0.045*
	Fusiform gyrus	Left	32	−27	−88	−10	0.031*
**Facial emotion processing: effect of group^e^**							
All	Area MT/V5	Right	145	54	−67	−4	0.001
	Middle temporal gyrus	Right	–	60	−55	11	–
	Fusiform gyrus	Right	32	42	−46	−16	0.020*

The table presents functional MRI correlates for the individual specified contrasts across the combined participant cohort (all groups). Voxel coordinates of local maxima within significant clusters are in standard MNI stereotactic space. *P*-values represent cluster-level FWE-corrected values over the whole brain, except *peak level FWE-corrected within pre-specified regions of interest.

Key contrasts were formed as follows.

^a^T contrast facial emotion > fixation cross; ^b^T contrast facial emotion > mosaic; ^c^T contrast positive emotion > negative emotion; ^d^T contrast negative emotion > positive emotion; ^e^F contrast facial emotion > mosaic.

**Table 3:** Functional neuroanatomical associations of emotion identification and physiological responses

**Group**	**Region**	**Side**	**Cluster (voxels)**	**Peak (mm)**			***P*_FWE_**
				***x***	***y***	***z***	
**Emotion identification performance (after covarying for semantic ability)**							
bvFTD	Anterior insula	Left	167	−24	29	11	0.009
	Caudate	Left	–	−15	11	8	–
svPPA	Temporal pole	Right	4	36	2	−37	0.015*
nfvPPA	Frontal operculum	Right	100	48	11	11	0.023*
**Cardiac parasympathetic activity^a^**							
svPPA	Fusiform gyrus	Left	166	−36	−28	−16	0.008
	Middle temporal gyrus	Left	142	−57	−49	−16	0.019
	Superior frontal gyrus	Left	131	−18	−1	68	0.028
	Fusiform gyrus	Right	49	18	−76	−16	0.033*
nfvPPA	Dorsolateral prefrontal	Right	3023	36	38	17	<0.001
	Medial prefrontal	Right	–	18	22	49	–
		Left	–	−6	42	33	–
	Anterior cingulate	Right	–	10	45	11	–
		Left	–	−10	48	16	–
	Caudate	Left	–	−9	2	14	–
	Insula	Right	–	43	2	−2	–
	Frontal operculum	Left	343	−42	20	8	<0.001
	Superior temporal sulcus	Right	122	48	−34	1	0.040
**Cardiac sympathetic activity^b^**							
nfvPPA	Orbitofrontal cortex	Right	346	15	5	−19	<0.001
	Temporoparietal junction	Right	160	45	−34	29	0.010
	Pons	Right	119	1	−25	−32	0.045
	Lateral medulla	Right	–	14	−29	−44	–
		Left	–	−7	−27	−44	–
	Insula	Left	76	−36	−1	−13	<0.001*
**Pupil activity**							
svPPA	Angular gyrus	Left	186	−42	−64	59	0.004
		Right	122	45	−40	41	0.039
	Fusiform gyrus	Left	129	−18	−52	−7	0.030
		Right	37	42	−67	−16	0.017*
	Temporal pole	Left	68	−27	14	−31	0.001*
nfvPPA	Anterior cingulate	Right	62	12	17	23	0.045*

The table presents functional MRI correlates for the specified response measures at second level within each syndromic group. Voxel coordinates of local maxima within significant clusters are in standard MNI stereotactic space. *P*-values represent cluster-level FWE-corrected values over the whole brain, except *peak-level FWE-corrected within pre-specified regions of interest.

^a^Negative association with heart rate change.

^b^Positive association with heart rate change.

Across the combined participant cohort, early visual processing (video>fixation cross condition) was associated with bi-hemispheric activation of primary visual cortex, while facial emotion-specific sensory processing (facial emotion > scrambled mosaic condition) was associated with bi-hemispheric activation of fusiform face area (Kanwisher *et al.*, 1997) and a cluster of association cortices including MT/V5 (Dumoulin *et al.*, 2000), angular gyrus, posterior superior temporal sulcus and middle temporal gyrus (Fig. 1). Valence-specific contrasts revealed greater activation of early visual processing areas by positive emotions (bilateral cuneus; positive emotion > negative emotion contrast), and greater activation of higher visual processing areas associated with face and biological motion detection by negative emotions (bilateral fusiform, right lingual gyrus and MT/V5; negative emotion>positive emotion contrast).

Activation of primary visual cortex did not differ between participant groups. However, activation of right fusiform and temporo-occipital junctional cortices showed a main effect of participant group: beta plots (Fig. 2) revealed reduced posterior middle temporal gyrus activation relative to healthy controls in the bvFTD and nfvPPA groups, and reduced fusiform activation relative to healthy controls in all syndromic groups.

Activations predicting facial emotion identification performance after covarying for semantic competence were found in syndrome-specific loci (Fig. 3): for the bvFTD group, left anterior insula and caudate; for the svPPA group, right temporal polar cortex; and for the nfvPPA group, right frontal operculum.

Complex syndromic activation profiles correlating with autonomic reactivity were identified (Figs 4 and 5). Within the svPPA group, cardiac deceleration (reflecting parasympathetic activity) was associated with activation of fusiform gyrus bilaterally, left middle temporal and superior frontal gyri; while pupil dilatation was associated with activation of fusiform and angular gyri bilaterally and left temporal pole. Within the nfvPPA group, cardiac deceleration was associated with activation of medial prefrontal cortex bilaterally, right superior temporal sulcus, insula and anterior cingulate and left frontal operculum; while cardiac acceleration (reflecting sympathetic activity) was associated with activation of right temporo-parietal junction and orbitofrontal cortex, left insula and brainstem (central pons in the vicinity of locus coeruleus, parabrachial complex and ventrolateral medulla); and pupil dilatation was associated with activation of right anterior cingulate. No significant associations of autonomic reactivity were identified in the healthy control or bvFTD groups at the prescribed threshold.

## Discussion

Here we have shown that canonical FTD syndromes have functional neuroanatomical signatures across three core dimensions of facial emotion processing—perceptual decoding, explicit categorization and autonomic arousal. These signatures map onto the hierarchical network architecture implicated in the processing of socio-emotional signals in the healthy brain (Alcalá-López *et al.*, 2017).

Despite consistent activation of primary visual cortex (Fig. 1), activation of fusiform and occipito-temporal junctional cortices was differentially attenuated across FTD syndromic groups (Fig. 2). In the healthy brain, fusiform gyrus and area MT/V5 participate in a ‘visual-sensory’ processing network (Alcalá-López *et al.*, 2017) that encodes facial expressions and other dynamic signals (Vuilleumier *et al.*, 2001; Kilts *et al.*, 2003; Pelphrey *et al.*, 2007; Haxby and Gobbini, 2011; Foley *et al.*, 2012) while posterior middle temporal gyrus (together with superior temporal sulcus) is a multimodal hub linking encoding of dynamic stimulus features to higher-order associative processes such as behavioural context and theory of mind (Said *et al.*, 2010; Deen *et al.*, 2015; Alcalá-López *et al.*, 2017; Schuwerk *et al.*, 2017; Ballotta *et al.*, 2018). In line with previous evidence in the healthy brain (Fusar-Poli *et al.*, 2009), our data further demonstrate emotion specificity at the level of visual analysis, reflecting the neural resources required to differentiate the valence of facial expressions: positively-valenced (smiling) faces can be distinguished perceptually from other expressions based on elementary configurational feature decoding in early visual areas, whereas differentiation of negatively-valenced facial expressions demands a more fine-grained categorical analysis, engaging higher order visual cortices (fusiform gyrus and MT/V5).

Our findings extend previous work in bvFTD (Virani *et al.*, 2013; De Winter *et al.*, 2016), demonstrating that nfvPPA (but not svPPA) is also associated with reduced engagement of the temporo-occipital hub for dynamic facial expression processing while all major FTD syndromes lead to reduced activity in fusiform face-responsive cortex. Moreover, visual cortical responses were not the key drivers of emotional identification performance. Consistent with previous work (Hutchings *et al.*, 2017), this was impaired across FTD syndromes but predicted by syndrome-specific activation of more anterior cortical regions linked to visual association areas (Alcalá-López *et al.*, 2017) (Fig. 3): anterior insula and caudate in bvFTD, anterior temporal cortex in svPPA and frontal operculum in nfvPPA. These distinct neuroanatomical associations suggest that the mechanism of impaired emotion categorization may differ between syndromes and arise at different levels of the processing hierarchy (Alcalá-López *et al.*, 2017). Emotion identification in the bvFTD and nfvPPA groups was driven by activation of intermediate-integrative network elements: anterior insula plays a key role in integrating body state representations and affective judgements (Jabbi and Keysers, 2008; Craig, 2009), while both caudate and frontal operculum have been implicated in motoric processing of dynamic emotional faces, providing a substrate for ‘mirror’ activity supporting empathic emotion identification (Montgomery *et al.*, 2009; Said *et al.*, 2010; Trinkler *et al.*, 2017). In contrast, emotion identification in the svPPA group was determined by a core element of the higher associative network in right anterior temporal lobe, which instantiates social concepts and person-specific semantics (Zahn *et al.*, 2007; Olson *et al.*, 2013).

The autonomic findings here amplify mounting evidence for central autonomic dysregulation in FTD (Joshi *et al.*, 2014, 2017; Fletcher *et al.*, 2015*b*; Guo *et al.*, 2016; Marshall *et al.*, 2017, 2018*a*). The neuroanatomical associations of cardiac responses here conformed broadly to the partitioning of cerebral sympathetic and parasympathetic regulatory mechanisms in previous studies of the healthy brain (Beissner *et al.*, 2013). Cardiac parasympathetic reactivity to facial emotions was impaired in all FTD syndromes, while pupil reactivity was impaired in nfvPPA. Our neuroanatomical findings support distinct mechanisms of altered autonomic reactivity in svPPA and nfvPPA. In the svPPA group, this was mediated by fusiform together with posterior temporo-parietal, temporal polar and prefrontal cortices, previously linked to parasympathetic autonomic responses and pupillary visuomotor tracking (Critchley *et al.*, 2005; Beissner *et al.*, 2013; Hosseini *et al.*, 2017); while in the nfvPPA group, autonomic responses were mediated by cingulo-insular and inferior frontal integrative and higher-order dorsal fronto- and temporo-parietal associative areas conjointly with brainstem sympathetic and parasympathetic pathways (Critchley *et al.*, 2005; Beissner *et al.*, 2013; Alcalá-López *et al.*, 2017). The lack of a group-level functional neuroanatomical correlate of cardiac hyporeactivity for the bvFTD group may reflect the pathological and structural neuroanatomical heterogeneity of this syndrome (Warren *et al.*, 2013*a*).

Whereas all three FTD syndromes were associated with impaired explicit identification of facial expressions and reduced engagement of face-responsive fusiform cortex, their distinctive syndromic profiles of higher-order evaluative and autonomic dysfunction corroborate previous studies of neural network organization in the healthy brain (Critchley *et al.*, 2005; Beissner *et al.*, 2013; Alcalá-López *et al.*, 2017). In bvFTD, core network dysfunction centred on middle temporal gyrus, anterior insula and dorsal striatum: regions integral to the integration of bodily and relevant environmental signals with output behaviours, including mental state judgments. This is in line with previous evidence for profoundly disturbed emotional mimesis and homeostatic signalling in this syndrome (Marshall *et al.*, 2018*a*, *b*) and also with the cardiac parasympathetic deficit here. In svPPA, core network dysfunction centres on areas (notably, anterior temporal cortex) involved in appraisal of salient socio-emotional and other environmental stimuli, and implicated both in emotion categorization and autonomic reactivity (Fletcher *et al.*, 2015*b*, 2016). In nfvPPA, core frontal opercular dysfunction underpins deficits of both cognitive and autonomic emotional responses, embedded in a distributed cortico-subcortical signature of autonomic dysregulation that extends to brainstem effector circuitry: this aligns with previous evidence for autonomic hyporeactivity in nfvPPA (Fletcher *et al.*, 2015*a*, *b*; Marshall *et al.*, 2018*a*). Moreover, the syndrome of nfvPPA is often a variant of progressive supranuclear palsy (Josephs and Duffy, 2008), with associated midbrain atrophy. This is one possible explanation for the selective loss of pupil reactivity in this syndrome but requires further work to confirm (Fletcher *et al.*, 2016).

This work has several limitations and raises a number of important issues for future clarification. Larger patient cohorts with histopathological and genetic correlation will be required to define the pathophysiological phenotypes delineated here fully (Perry *et al.*, 2017). The interpretation of BOLD signal changes in neurodegenerative disease cohorts is complicated by the presence of grey matter atrophy: it is noteworthy that a number of functional neuroanatomical associations in the present cohort fell outside regional atrophy zones for these syndromes (Supplementary Fig. 1) but on the other hand, certain structures that are integral to emotion processing—notably, amygdala—were conspicuously absent here. This might be attributable (at least in part) to reduced BOLD signal due to severe atrophy but could also reflect the nature of the paradigm. Engagement of amygdala may require stimuli to carry emotional or other behavioural significance for the viewer (Strathearn and Kim, 2013; Kumfor *et al.*, 2018): our facial expression stimuli were relatively banal. This raises the broader issue of paradigm design: an in-scanner task with manipulation of behavioural context would likely modulate activation profiles (Alcalá-López *et al.*, 2017; Kumfor *et al.*, 2018), and indeed, the separable correlates of emotion perception and identification here hint at such a modulatory effect. The absence of a correlated task might also account for the finding of impaired cardiac reactivity in svPPA, in contrast to previous observations (Marshall *et al.*, 2018*a*).

## Conclusions

Our findings in the working brain in FTD suggest a refinement of the influential neural network paradigm of these diseases (Warren *et al.*, 2013*b*; Perry *et al.*, 2017): rather than a unitary mapping between clinical phenotype and brain network dysfunction, we have demonstrated coactivation of distributed sensory and associative networks across FTD syndromes (Alcalá-López *et al.*, 2017). A further key emerging theme in FTD and related neurodegenerative diseases is the centrality of homeostatic dysfunction to socio-emotional symptoms (Cersosimo and Benarroch, 2013; Marshall *et al.*, 2017; Trinkler *et al.*, 2017; Ahmed *et al.*, 2018; Marshall *et al.*, 2018*a*, *b*): integration of functional MRI with simultaneous autonomic recordings here has underscored this, by revealing a rich matrix of central autonomic dysregulatory changes overlapping network profiles of emotional visual and categorization processing in FTD syndromes. An important direction for further work will be to define more precisely the relative contributions of aberrant stimulus decoding and primary failure of central autonomic control to diminished physiological reactivity in different FTD syndromes. The use of naturalistic, dynamic emotional stimuli (as here) is likely to be critical to delineate homeostatic processes that evolve over time; this might also motivate the application of functional neuroimaging techniques such as magnetoencephalography with high temporal resolution (Macey *et al.*, 2015; Hughes *et al.*, 2018).

This work has far-reaching clinical as well as pathobiological implications. Functional neuroimaging can reveal disease effects beyond and predating the development of irreversible network degeneration, including presymptomatic changes in genetic cases (Rohrer *et al.*, 2015). More fundamentally, work of this kind promises to deliver a new class of pathophysiological dementia biomarkers: if, as our findings suggest, autonomic measures are surrogates for complex socio-emotional behaviours and neural network dysfunction, this could find powerful applications in early diagnosis, disease tracking and the evaluation of new therapies.

## Supplementary Material

awz204_Supplementary_Data

## Abbreviations

(bv)FTD: (behavioural variant) frontotemporal dementia
nfv/svPPA: non-fluent variant/semantic variant primary progressive aphasia
