# Supplementary material for: The functional neuroanatomy of emotion processing in frontotemporal dementias
Source: Brain. 2019 Jul 18;142(9):2873–87. doi: 10.1093/brain/awz204 (PMC7959336; doi:10.1093/brain/awz204)
Supplement: awz204_Supplementary_Data [file awz204_supplementary_data.pdf]

The functional neuroanatomy of emotion processing in frontotemporal dementias

Charles R Marshall[1,2,3], Christopher JD Hardy[2], Lucy L Russell[2], Rebecca L Bond[2], Harri Sivasathiaseelan[2], Caroline Greaves[2], Katrina M Dick[2], Jennifer L Agustus[2], Janneke EP van Leeuwen[2], Stephen J Wastling[4], Jonathan D Rohrer[2], James M Kilner[3], Jason D Warren[2]

[1]Preventive Neurology Unit, Wolfson Institute of Preventive Medicine, Queen Mary University of London

[2]Dementia Research Centre, Department of Neurodegenerative Disease, UCL Institute of Neurology

[3]Sobell Department of Motor Neuroscience and Movement Disorders, UCL Institute of Neurology

[4]Department of Neuroradiology, National Hospital for Neurology and Neurosurgery

London United Kingdom

Corresponding author: Dr Charles Marshall, Preventive Neurology Unit, Wolfson Institute of Preventive Medicine, Queen Mary University of London, London, UK EC1M 6BQ. Email: charles.marshall@qmul.ac.uk, tel: 0207 882 3528.

Supplementary Table 1: Medication use in participant groups

Medication	Control	bvFTD	svPPA	nfvPPA
ACE inhibitor	6	1	2	0
Ca blocker	3	1	0	0
Diuretic	2	2	0	1
Antihistamine	2	1	1	1
Statin	5	5	3	3
Antidepressant	2	8	5	5
Levothyroxine	1	1	2	2
Proton pump inhibitor	1	1	2	3
Nitrate	1	0	1	0
Beta blocker	0	2	1	1

The table shows the number of participants in each group taking each medication type. Control, healthy control group; bvFTD, patients with behavioural variant frontotemporal dementia; svPPA, patients with semantic variant primary progressive aphasia; nfvPPA, patients with nonfluent variant primary progressive aphasia.

Supplementary figure 1: Atrophy distribution in patient groups

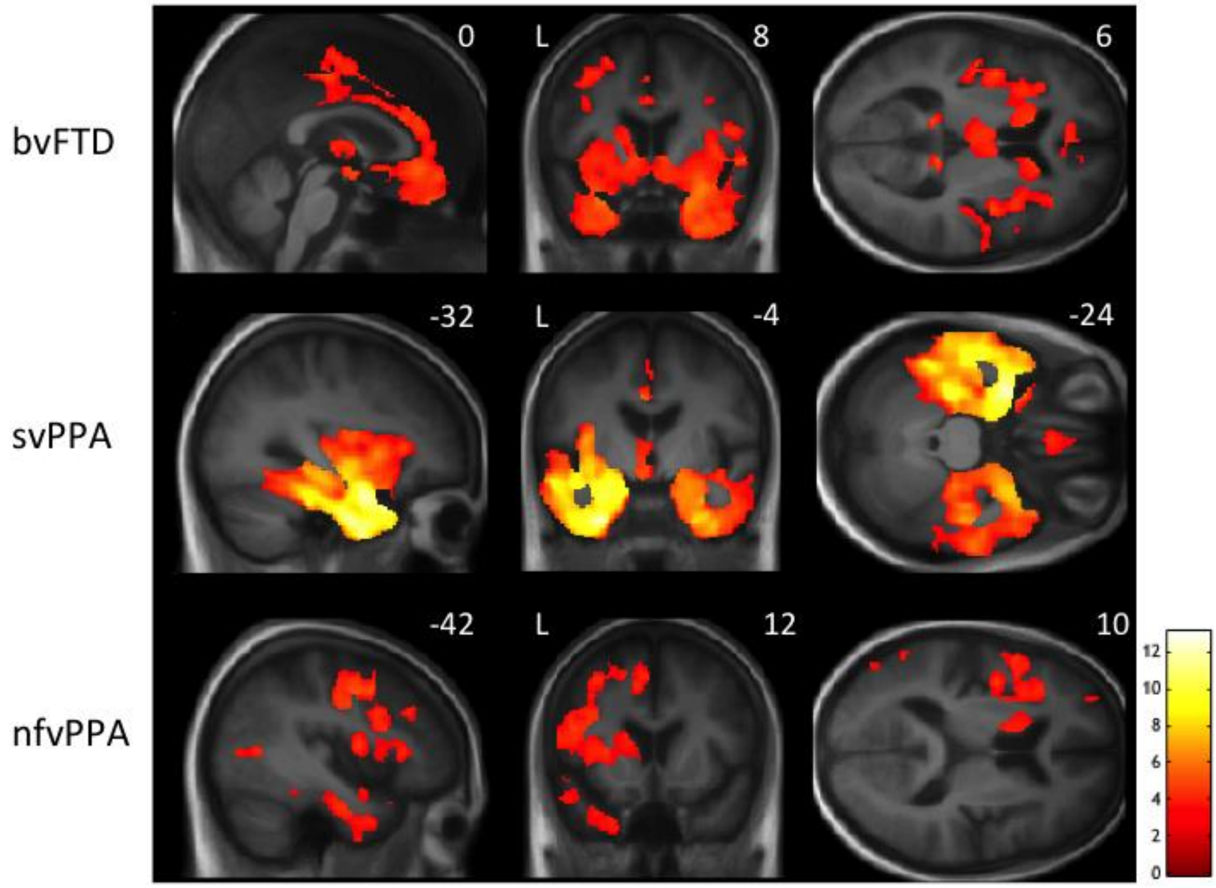

The figure shows statistical parametric maps of atrophy distribution in each patient group derived from voxel based morphometry (see Supplementary Methods for details). SPMs are thresholded at $p<0.001$ uncorrected and displayed on sections of the structural group mean T1-weighted template brain image. The plane of each section (in mm in MNI space) is shown in the top right of each image; coronal sections show the right hemisphere on the right. The colour bar codes T values. bvFTD, patient group with behavioural variant FTD; nfvPPA, patient group with nonfluent variant primary progressive aphasia; svPPA, patient group with semantic variant primary progressive aphasia.

Supplementary Figure 2: Mean control subject heart rate response

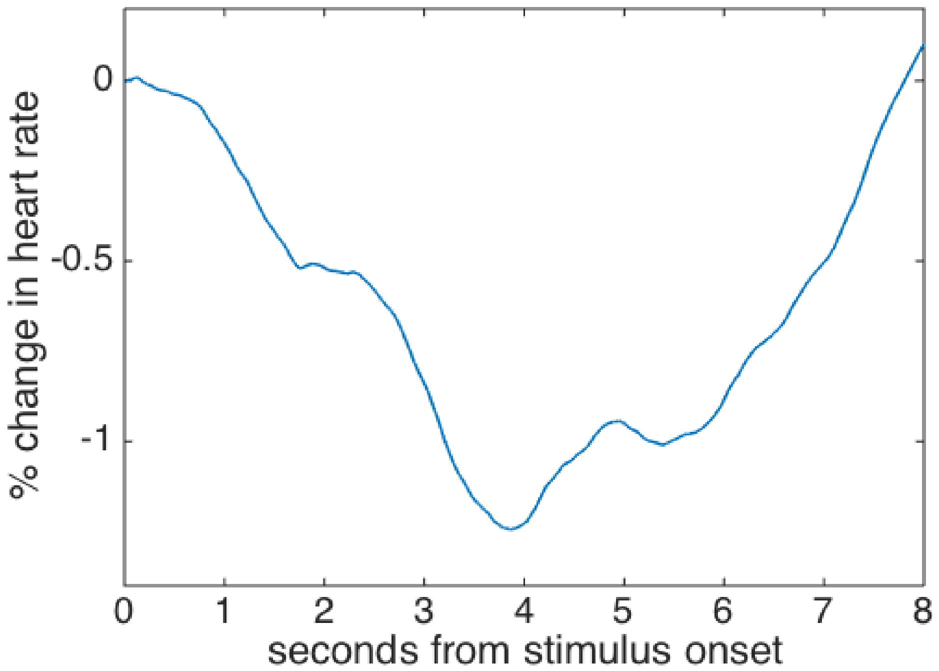

The figure shows the mean smoothed heart rate response for all healthy control subjects for facial emotion trials (see Methods for details).

Supplementary Figure 3: Heart rate response by stimulus type in each participant group

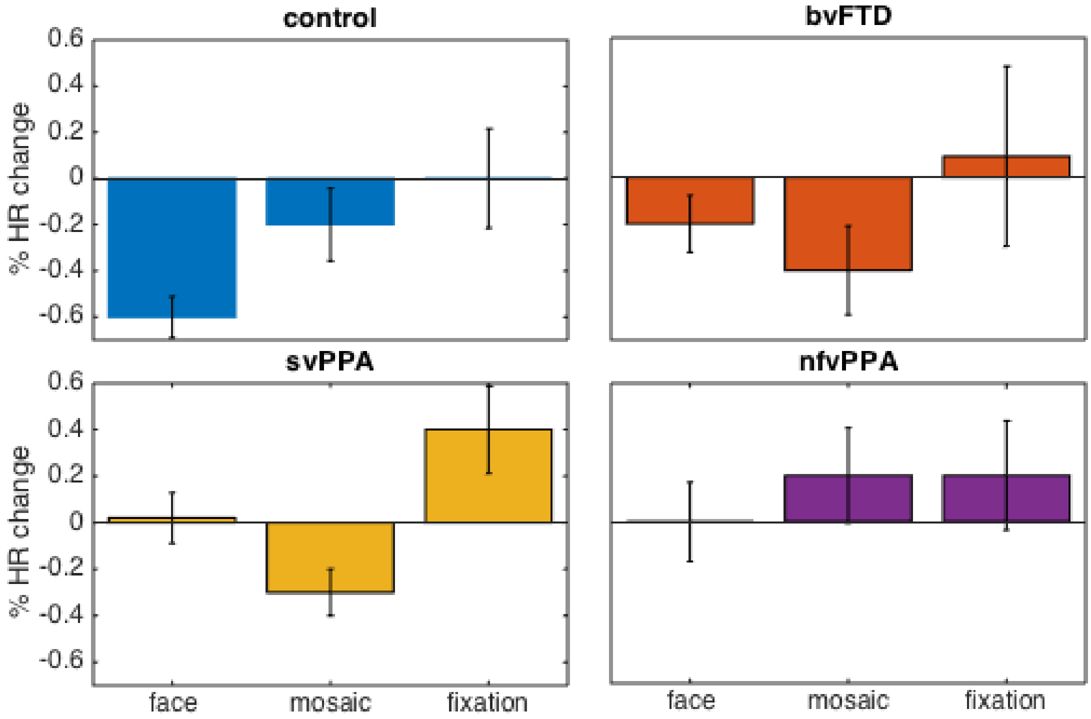

The figure shows mean percentage change in heart rate for each stimulus type, separately for each diagnostic group. Error bars represent the standard error of the mean. bvFTD, patient group with behavioural variant frontotemporal dementia; control, healthy control group; face, dynamic facial emotion trials; fixation, fixation cross trials; HR, heart rate; mosaic, dynamic mosaic trials; nfvPPA, patient group with nonfluent variant primary progressive aphasia; svPPA, patient group with semantic variant primary progressive aphasia.

Supplementary Figure 4: Pupil response by stimulus type in each participant group

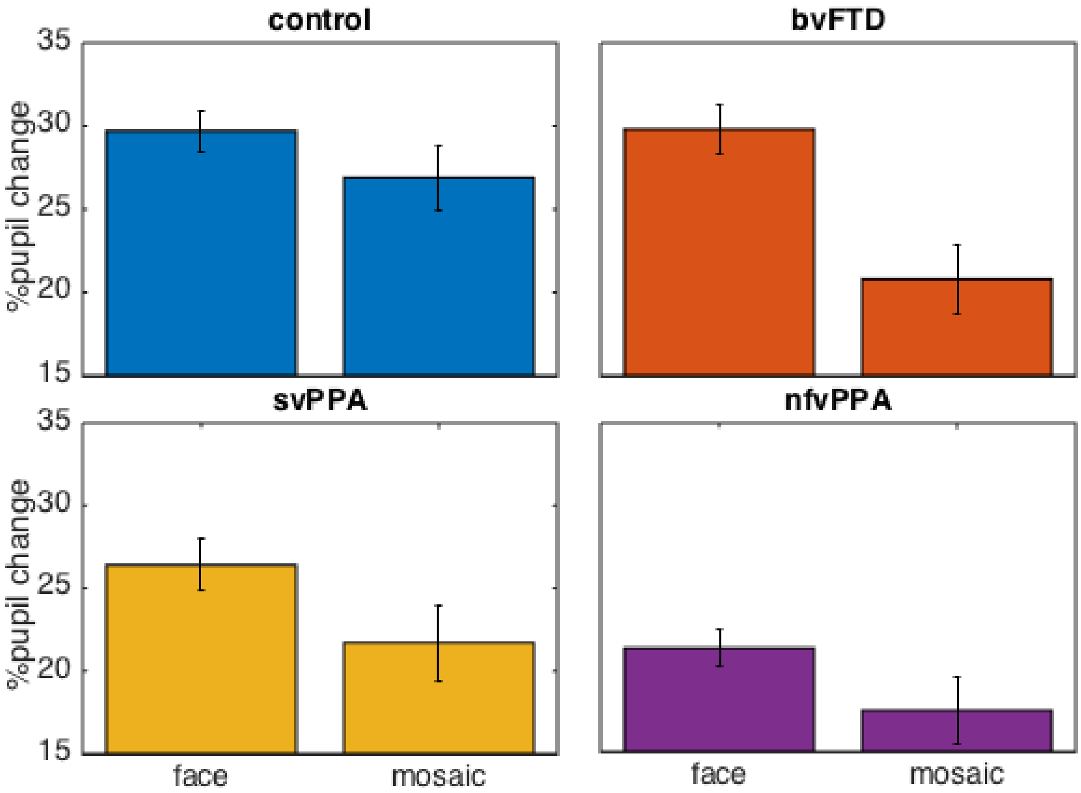

The figure shows mean percentage change in pupil size for each stimulus type, separately for each diagnostic group. Fixation cross trials are not shown as the difference in luminance makes them unsuitable for direct comparison. Error bars represent the standard error of the mean. bvFTD, patient group with behavioural variant frontotemporal dementia; control, healthy control group; face, dynamic facial emotion trials; mosaic, dynamic mosaic trials; nfvPPA, patient group with nonfluent variant primary progressive aphasia; svPPA, patient group with semantic variant primary progressive aphasia.

Supplementary methods:

VOXEL BASED MORPHOMETRY ANALYSIS

A volumetric T1 brain image was acquired for each participant as described in Methods. These were used to assess the distribution of grey matter atrophy in each patient group in a voxel based morphometry (VBM) analysis. Pre-processing was performed using the New Segment and DARTEL toolboxes of SPM8 (www.fil.ion.ucl.ac.uk/spm), following an optimised protocol. Normalisation, segmentation and modulation of grey and white matter images were performed using default parameter settings and grey matter images were smoothed using a 6 mm full width-at-half-maximum Gaussian kernel. A study-specific template mean brain image was created by warping all bias-corrected native space brain images to the final DARTEL template and calculating the average of the warped brain images. Total intracranial volume was calculated for each participant by summing grey matter, white matter and cerebrospinal fluid volumes after segmentation of tissue classes. Processed brain MR images were entered into a VBM analysis of the entire participant cohort. A full factorial model with participant group as a level variable was used to demonstrate voxels that were smaller in volume in each patient group relative to the healthy control group. Age and total intracranial were incorporated as covariates of no interest in the model. To visualize the distribution of atrophy in each group, statistical parametric maps were assessed at a threshold of $p<0.001$ uncorrected (see Supplementary Figure 1).

Performance of SPM preprocessing in the presence of severe atrophy

In the presence of severe atrophy, there is a risk of inappropriately excluding voxels from analysis in regions most affected by atrophy, thereby resulting in false negatives in imaging analysis (Ridgway *et al.*, 2009). One would expect this issue to be most problematic in the svPPA group, where marked temporal lobe atrophy is seen, often with severely atrophic 'knife blade' gyri (Marshall *et al.*, 2018c). We used careful visual quality control to ensure that no parts of atrophic temporal lobes were excluded in any of the preprocessing steps (see Supplementary Figure 5 for an example in a single subject from the svPPA group). At second level, we used an established method to ensure regions showing atrophy in some subjects were not excluded from analysis by using an explicit majority threshold mask rather than relying on implicit masking (Ridgway *et al.*, 2009).

Supplementary Figure 5: Visual quality control of SPM preprocessing in the presence of severe atrophy

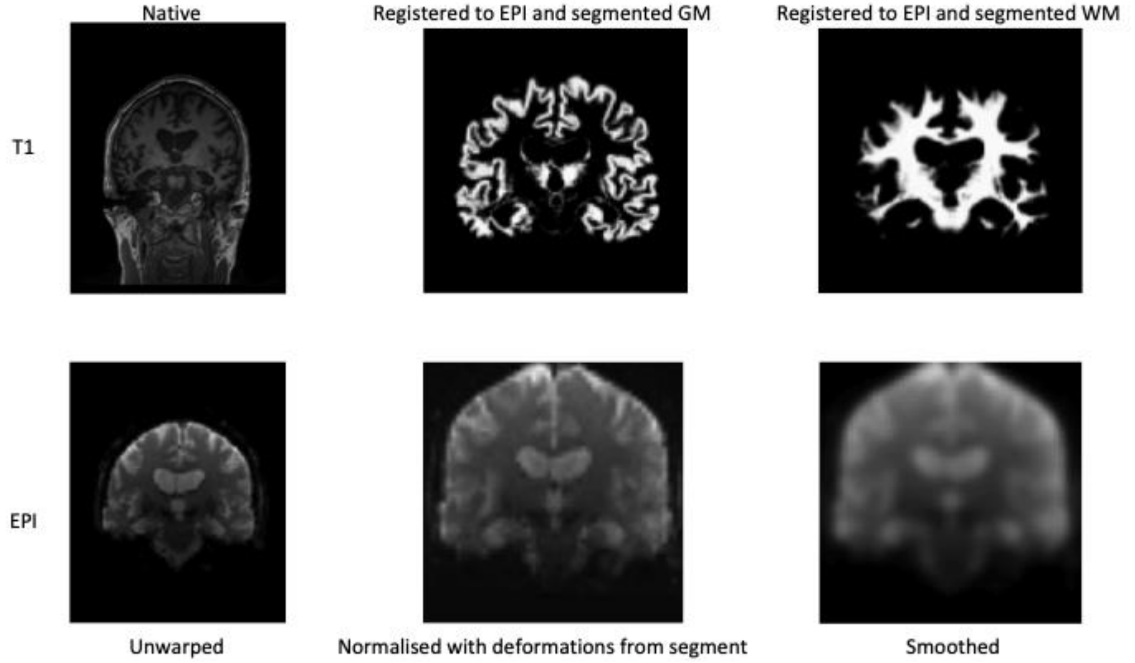

The figure shows representative coronal MRI sections of one of the participants in the svPPA group at key stages of SPM preprocessing. Native T1 images (top left) were registered to the unwarped EPI (bottom left) before segmentation into grey matter (top middle), white matter (top right) and CSF. The forward deformations from the segmentation were used to normalize the unwarped EPI (bottom middle), which was then smoothed with a 6mm full width at half maximum Gaussian kernel (bottom right).
